# Comparison of cough reflex sensitivity after an inhaled antigen challenge between actively and passively sensitized guinea pigs

**DOI:** 10.1186/1745-9974-1-6

**Published:** 2005-09-06

**Authors:** Johsuke Hara, Masaki Fujimura, Shigeharu Myou, Yoshitaka Oribe, Shiho Furusho, Toshiyuki Kita, Nobuyuki Katayama, Miki Abo, Noriyuki Ohkura, Yoriko Herai, Akihiro Hori, Yoshihisa Ishiura, Kouichi Nobata, Haruhiko Ogawa, Masahide Yasui, Kazuo Kasahara, Shinji Nakao

**Affiliations:** 1 Respiratory Medicine, Cellular Transplantation Biology, Kanazawa University Graduate School of Medical Science, 13-1, Takara-machi, Kanazawa City, Ishikawa, 920-8641, Japan

## Abstract

**Background:**

Late asthmatic response is observed following antigen challenge in actively, but not passively, sensitized guinea pigs. Although cough reflex sensitivity is increased after antigen challenge in actively sensitized guinea pigs, it is unknown whether the antigen-induced increase in cough reflex sensitivity develops in passively sensitized animals. The aim of this study was to compare the cough reflex sensitivity to inhaled capsaicin after an inhaled antigen challenge between actively and passively sensitized guinea pigs.

**Methods:**

Measurement of number of coughs elicited by increasing concentrations of capsaicin (10^-6 ^and 10^-4 ^M) and bronchial responsiveness to ascending concentrations of methacholine, and analysis of bronchoalveolar lavage fluid (BALF) were separately performed 24 h after an antigen challenge in actively and passively sensitized guinea pigs.

**Results:**

Percentage of eosinophils in BALF and bronchial responsiveness to methacholine were increased 24 h after the antigen challenge in both actively and passively sensitized animals compared with saline-challenged actively and passively sensitized animals, respectively. Absolute number of eosinophils in BALF from actively sensitized and antigen-challenged guinea pigs was significantly greater than that from passively sensitized and antigen-challenged animals. Cough response to capsaicin and concentration of substance P in BALF were increased 24 h after the antigen challenge in actively sensitized guinea pigs, but not in passively sensitized guinea pigs. Bronchial responsiveness, cough reflex sensitivity and substance P concentration and total cells in BALF were increased in actively sensitized and saline challenged guinea pigs compared with passively sensitized and saline challenged animals.

**Conclusion:**

The results suggest that active sensitization per se increases cough reflex sensitivity accompanied by increased inflammatory cells and substance P level in BALF, and antigen challenge further increases them, while simple IgE- and/or IgG-mediated allergic reaction per se or the low intensity of eosinophil infiltration in the airway itself may not affect cough reflex sensitivity in guinea pigs.

## Background

Chronic cough is a common and distressing symptom. Eosinophilic airway disorders such as eosinophilic bronchitis without asthma [1] and atopic cough [2] are important causes of the chronic cough. In these disorders, cough reflex sensitivity is heightened while patients are coughing and becomes normal on successful treatment [3]. Knowledge of the detailed pathogenesis is needed to understand the mechanism and to develop better treatment of the disorders.

We have shown in actively sensitized guinea pigs that cough reflex sensitivity is increased 24 h after an inhaled antigen challenge, which is not mediated by bronchoconstriction [4]. Allergic reaction and cough hypersensitivity may be induced by chemical mediators such as histamine [5], prostaglandins [6], thromboxane A2 (TXA2) [4], and platelet activating factor (PAF), which are released from mast cells activated by IgE antibody and/or production of Th2 cytokines [7] such as IL-4, IL-5 and IL-13. On the other hand, simple IgE- and/or IgG-mediated allergic airway reaction occurs when passively sensitized guinea pigs are challenged with an aerosolized antigen. It is, however, unknown whether the simple IgE- and/or IgG-mediated allergic airway reaction can increase cough reflex sensitivity. To elucidate this, we compared the cough reflex sensitivity to inhaled capsaicin after an inhaled antigen challenge between actively and passively sensitized guinea pigs.

## Methods

### Animals

Male, albino, Hartley-strain guinea pigs were obtained from Sankyou Laboratory Service (Toyama, Japan). They were quarantined in the Animal Research Center of Kanazawa University. All the animal procedure in this study complied with the standards set out in the Guideline for the Care and Use of Laboratory Animals at the Takara – machi Campus of Kanazawa University.

### Study design

In order to avoid possible interaction between capsaicin-induced cough, methacholine-induced bronchoconstriction and BALF contents, measurement of cough reflex sensitivity to inhaled capsaicin, measurement of bronchial responsiveness to inhaled methacholine and BAL were separately carried out 24 hours after an aerosolized antigen challenge in actively and passively sensitized guinea pigs.

### Active sensitization and antigen challenge

Actively sensitized guinea pigs were assigned into two groups: saline challenge (A-OA/Sal) and OA challenge (A-OA/OA) groups (n = 8 for each group). Animals in A-OA/Sal group were challenged with aerosolized saline, and A-OA/OA group with aerosolized antigen. Guinea pigs weighing 200 to 220 g each were actively sensitized by the method reported by Muraki et al [8]. Animals were given an intraperitoneal administration of 2.0 mg of ovalbumin (OA) and 100 mg of aluminum hydroxide [Al(OH)_3_] 2 days after an intraperitoneal administration of 30 mg/kg cyclophosphamide. Three weeks later, boosting was carried out by intraperitoneal administration of 0.01 mg of OA and 100 mg of Al(OH)_3_. Three weeks after the boosting, actively sensitized guinea pigs were challenged with an aerosolized OA solution under spontaneous breathing at 20 min after an intraperitoneal administration of diphenhydramine (20 mg/kg) to avoid acute anaphylactic respiratory distress. Conscious guinea pigs were placed in a dual chamber plethysmograph (head chamber volume, 1520 ml) (model PMUA + SAR, Buxco Electronics, Sharon, CT). Animals were challenged with 10 mg/ml OA aerosol for 90 s (head chamber only, 0.08 ml/min output). The aerosol was generated by a Devilbiss 646 nebulizer (Devilbiss Co., Somerset, PA) operated by compressed air at 7.57 L/min (Minipon 54B-588, Origin Medical Industry Co., Ltd., Tokyo, Japan).

### Passive sensitization and antigen challenge

Guinea pig homocytotropic antiserum was obtained by the method elaborated in Santives et al. [9]. Briefly, 500 μg of ovalbumin (OA) was emulsified in Freund's complete adjuvant and injected intradermally into each guinea pig at multiple sites. A booster dose was prepared and administered in the same manner 2 weeks later. Serum collected from each animal 2 weeks after the booster dose was pooled, and kept frozen until use. The antibody titre of this serum was 1:12,800, 1:6,400 and 1:512, as estimated by passive cutaneous anaphylaxis at 4 h, 24, and 7 days, respectively. Normal guinea pigs were passively sensitized with 1.0 mL/kg antiserum intraperitoneally.

Passively sensitized guinea pigs weighing 450 to 500 g were assigned into two groups: saline challenge (P-OA/Sal) and OA challenge (P-OA/OA) groups (n = 8 for each group). Animals in P-OA/Sal group were challenged with aerosolized saline, and P-OA/OA group with aerosolized antigen. One week after the passive sensitization, guinea pigs were challenged with an aerosolized OA solution (10 mg/mL) under spontaneous breathing at 20 min after an intraperitoneal administration of diphenhydramine (20 mg/kg). OA challenge to passively sensitized guinea pigs was carried out by the same method used in actively sensitized model.

### Cough reflex sensitivity

Cough reflex sensitivity was measured 24 h after challenge with either OA or saline in both actively and passively sensitized guinea pigs. Each conscious guinea pig was placed in an airtight custom-built transparent plastic box consisting of a head chamber (1600 ml volume) isolated from a body chamber, and pressure in the body chamber was recorded. Coughs were detected as a change in the pressure (a rapid inspiration followed by rapid expiration). To disregard motion- and sneezing-related changes in the pressure, movements of the guinea pigs were visually monitored. Coughs were counted by a trained observer and recognized by the characteristic animal posture and the pressure transducer recordings. Increasing concentrations of capsaicin solution (10^-6^, 10^-4 ^M) were inhaled for 2 min from a Devilbiss 646 nebulizer (Devilbiss Co., Somerset, PA) operated by compressed air at 1.6 l/min (Iwaki Air Pump AP-115AN, Iwaki Co., Ltd., Tokyo, Japan). The nebulizer output was 0.037 ml/min. The number of coughs was counted during a 2 min inhalation of each capsaicin solution and for additional 1 min. The total number of coughs during the 3 – min period was recorded on the inhalation of each concentration of capsaicin.

### Bronchial responsiveness

Bronchial responsiveness to inhaled methacholine was measured 24 h after challenge with either OA or saline in both actively and passively sensitized guinea pigs. Guinea pigs were anesthetized by an intraperitoneal injection of 75 mg/kg of sodium pentobarbital and placed in a supine position. After the trachea was cannulated with a polyethylene tube (outside diameter, 2.5 mm; inside diameter, 2.1 mm), the animals were artificially ventilated using a small animal respirator (model 1680, Harvard Apparatus Co., Inc., South Natick, MA) adjusted to a tidal volume 10 ml/kg at a rate of 60 strokes/min. Ascending concentrations of methacholine solution (50, 100, 200, 400 μg/ml) were delivered for 20 s by an ultrasonic nebulizer (NE-U06, Omron, Kyoto, Japan) at 5 min intervals. The nebulizer generated the aerosol at a rate of 15.2 μl / min. The changes in lung resistance to insufflation, the lateral pressure of the tracheal tube (pressure at the airway opening abbreviated as Pao: cmH_2_O), were measured using a differential pressure transducer (model TP-603T, Nihon Koden Kogyo Co., Ltd., Tokyo, Japan). The change in Pao represents the average of the changes in pulmonary resistance (RL) and reciprocal dynamic lung compliance (1/Cdyn) [10].

### Bronchoalveolar lavage (BAL)

BAL was performed 24 h after challenge with either the antigen or saline in both actively and passively sensitized guinea pigs without capsaicin or methacholine provocation. Guinea pigs were anesthetized and prepared by the same method described in the measurement of bronchial responsiveness. Through the tracheal cannula the lungs were lavaged with 10 ml of saline 2 times (total: 20 ml). The cells in BAL fluid (BALF) were stained with Turk solution and counted in duplicate in a hemocytometer (in a Burker chamber). Differential cell counts were made on a smear prepared by cytocentrifuge and stained with Wright-Giemsa.

The concentration of substance P in BALF was measured using a commercial enzyme immunoassay (EIA) kit (Cayman Chemical Company, USA). This kit is a competitive assay that provides accurate measurements of substance P with a working range of 3.9 to 500 pg/ml.

### Preparation of drugs

The following chemicals were used: sodium pentobarbital (Abbott Laboratories, North Chicago, IL), methacholine (Wako Pure Chemical Ind., Osaka, Japan), diphenhydramine (Wako Pure Chemical Ind.), ovalbumin (Sigma, St. Louis, MO), Al(OH)_3 _(Wako Pure Chemical Ind.), dimethyl sulfoxide (Wako Pure Chemical Ind.), physiological saline (Otsuka Pharmaceutical Co., Ltd., Osaka, Japan), capsaicin (Sigma), cyclophosphamide (Shionogi Co., Ltd., Osaka, Japan).

### Statistical analysis

All data are shown as mean ± standard error of the mean (SEM). Statistical differences were determined by analysis of variance (ANOVA) followed by Fisher's protected test significant differences (Statview; SAS Institute, Cary, NC, USA). A *P *value less than 0.05 was considered statistically significant.

## Results

### Cough reflex sensitivity

**Figure 1:**
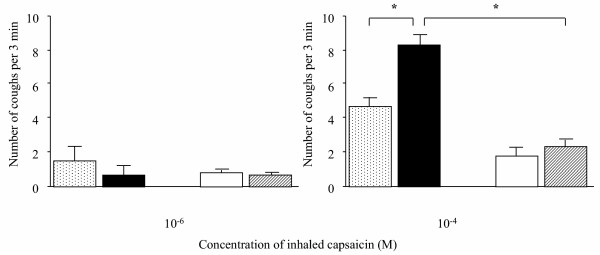


Fig. 1 shows the number of coughs induced by inhaled capsaicin in actively and passively sensitized guinea pigs. The number of coughs elicited by an aerosol of capsaicin (10^-4 ^M) was significantly increased in A-OA/OA group (8.3 ± 0.9), but not in P-OA/OA group (2.3 ± 0.8), compared with each saline-challenged group (A-OA/Sal; 4.8 ± 0.6, P-OA/Sal; 1.8 ± 0.7).

### Bronchial responsiveness

**Figure 2:**
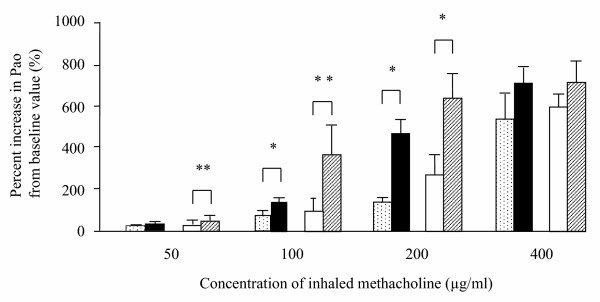


Bronchial responsiveness to inhaled methacholine in actively and passively sensitized guinea pigs are shown in Fig. 2. In the both groups, pressure at the airway opening (Pao) was dose-dependently increased by methacholine. The bronchial responsiveness in A-OA/OA (Percent increase in Pao from baseline value; 20.1 ± 16.5 %, 180.1 ± 30.5 %, 479.4 ± 89.2 %, 709.3 ± 99.8 % in 50, 100, 200, 400 μg/ml of inhaled methacholine) and P-OA/OA (51.1 ± 19.7 %, 364.7 ± 141.5 %, 637.4 ± 119.9 %, 717.2 ± 100.8 % in each concentration of methacholine) group was significantly heightened when compared with that in A-OA/Sal (5.5 ± 2.7 %, 87.2 ± 29.3 %, 182.9 ± 35.5 %, 529.1 ± 110.2 % in each concentration of methacholine) and P-OA/Sal (6.1 ± 2.8 %, 97.2 ± 61.4 %, 272.3 ± 94.5 %, 596.8 ± 64.2 % in each concentration of methacholine) group, respectively.

### BALF analysis

The percentage of eosinophils in BALF was significantly increased in both A-OA/OA and P-OA/OA group compared with A-OA/Sal and P-OA/Sal group, respectively. The total number of cells and eosinophils in BALF collected from A-OA/OA group were significantly increased compared with those from A-OA/Sal and P-OA/OA groups. The number of eosinophils in BALF collected from P-OA/OA group was significantly increased compared with those from P-OA/Sal group. There was no significant difference in the total number of cells between P-OA/OA and P-OA/Sal groups (Table 1).

**Table 1:** BAL fluid cell findings 24 h after an antigen inhalation in guinea pigs.

	Absolute number (cells/mL)				Percentage (%)				
	Total cells (10^3^)	Nac (10^3^)	Neu (10^3^)	Lym (10^3^)	Eos	Mac	Neu	Lym	Eos

AP group	201.0 ± 70.9*#	73.8 ± 18.7#	3.9 ± 1.5	3.3 ± 1.7#	120.0 ± 49.8*#	39.8 ± 4.8*#	2.1 ± 0.8	1.4 ± 0.3	56.7 ± 4.4*#
AN group	123.0 ± 28.8	93.2 ± 18.7	1.3 ± 1.1	2.6 ± 1.3	26.0 ± 11.7	77.9 ± 5.4	1.1 ± 0.9	1.9 ± 0.6	19.1 ± 6.3
PP group	60.5 ± 14.5	37.5 ± 3.1	2.1 ± 0.6	1.1 ± 0.2	19.9 ± 2.4$	61.6 ± 3.1$	3.9 ± 1.2	1.8 ± 0.4	32.3 ± 2.9$
PN group	57.6 ± 17.5	47.0 ± 5.2	1.8 ± 1.1	0.9 ± 0.2	7.9 ± 1.6	81.1 ± 2.4	3.8 ± 2.6	1.7 ± 0.5	13.4 ± 2.4

OA; ovalbumin, Sal: saline, A-OA/OA; OA inhalation in actively sensitized animals, A-OA/Sal; saline inhalation in actively sensitized animals, P-OA/OA; OA inhalation in passively sensitized animals, P-OA/Sal; saline inhalation in passively sensitized animals, Mac; macrophages, Neu; neutrophils, Lym; lymphocytes, Eos; eosinophils.

*P < 0.01 compared with the A-OA/Sal, #*P *< 0.01 compared with the P-OA/OA group, $*P *< 0.01 compared with the P-OA/Sal group.

**Figure 3:**
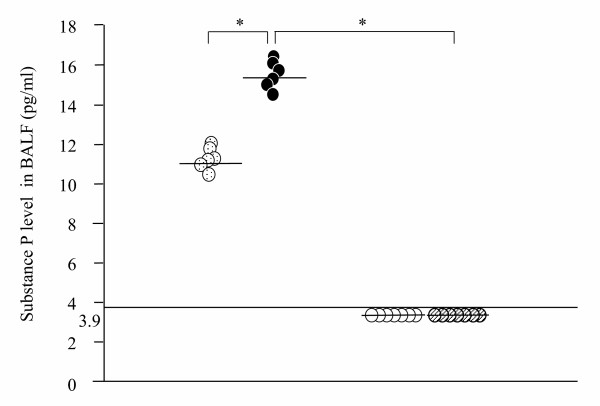


Fig. 3 shows the concentration of substance P in BALF. The concentration of substance P was significantly increased in A-OA/OA (15.9 ± 1.6 pg/ml) group compared with A-OA/Sal group (11.5 ± 1.2 pg/ml). The concentrations of substance P in P-OA/OA and P-OA/Sal groups were lower than 3.9 pg/ml.

## Discussion

The present study confirmed other researchers' investigation that active sensitization per se induces airway eosinophilic inflammation and increase in cough reflex sensitivity [11] and our previous data [5] that an aerosolized antigen challenge further enhances the airway responses in actively sensitized animals. We showed for the first time that cough reflex sensitivity was unchanged following an antigen challenge in passively sensitized guinea pigs while BAL eosinophils and bronchial responsiveness to methacholine were increased compared with saline challenged animals. In addition, substance P level in BAL fluid was increased in actively sensitized guinea pigs and further increased after an antigen challenge, but the level was below that measured in passively sensitized animals in spite of antigen challenge. These findings suggest that antigen-antibody reaction in the airway is insufficient to modulate cough reflex sensitivity. In other words, airway inflammatory processes such as cell and mediator response following antigen-antibody reaction may be important in increasing cough reflex sensitivity associated with increased levels of substance P.

Although BAL eosinophils and bronchial responsiveness were increased after antigen challenge in passively sensitized guinea pigs, cough reflex sensitivity and substance P levels in BAL fluid were unchanged. Airway eosinophil infiltration may not be essential in increasing cough reflex sensitivity. We previously reported that cough reflex sensitivity was not increased in patients with cough variant asthma complaining of daily coughing [3] and stable asthmatics [12], in both of whom eosinophilic airway inflammation is characteristic. Furthermore, Minoguchi et al. [13] suggested that cough reflex sensitivity to capsaicin is not associated with eosinophilic inflammation of the airway in patients with allergic asthma because antigen challenge did not influence cough reflex sensitivity to capsaicin. On the other hand, we have shown that challenge with environmental fungal antigen causes symptomatic cough accompanied by an increase in cough reflex sensitivity in patients with atopic cough [14-18]. We do not know why antigen challenge increases cough reflex sensitivity in atopic cough, but not in asthma. At least, airway allergic reactions other than airway eosinophil infiltration may be involved in increasing cough reflex sensitivity accompanied by an increase in substance P levels in the airway. The detailed mechanism should be disclosed in further studies.

In the present study, the concentration of substance P in BALF was increased 24 h after an antigen challenge in actively sensitized guinea pigs, but not in passively sensitized animals. Substance P has been considered as an important neuropeptide in the cough reflex pathway because tachykinin antagonists partially block the cough reflex. Neutral endopeptidase (NEP) has been recognized as the major enzyme degrading substance P [19]. We previously reported that NEP activity in tracheal tissue was decreased after an antigen challenge and the antigen-induced NEP inactivation might increase the cough response to capsaicin in actively sensitized guinea pigs [20]. In this respect, it is likely that the retention of NEP activity might be responsible for the lack of development of antigen-induced cough hypersensitivity in passively sensitized guinea pigs. Further studies are needed to clarify this possibility.

The receptor for capsaicin, termed vanilloid receptor-1 (VR-1), is expressed in guinea pigs. VR-1 mediates cough induced by capsaicin [21]. An increased expression of VR-1 has also been reported in humans with chronic cough [22]. In the airway of the guinea pig, VR-1 has been shown to be activated by a decrease in pH [23]. Recently, we reported that the pH of BALF was decreased in actively sensitized guinea pigs [24]. Therefore, in the airways of actively sensitized guinea pigs, but not of passively sensitized guinea pigs, the acid environment or epithelial damage might induce an increase in the number of VR-1 contributing to the enhanced cough reflex sensitivity. This possibility should be examined in future studies.

Simple IgE- and/or IgG-mediated allergic reactions induce eosinophilic infiltration in the airway and bronchial hyperresponsiveness to methacholine, but not cough hypersensitivity to capsaicin, in passively sensitized animals. The same difference between active and passive sensitization of animals is investigated concerning the antigen-induced late asthmatic response (LAR): LAR develops in actively, but not passively, sensitized guinea pigs [25]. It is suggested that the simple IgE- and/or IgG-mediated allergic reaction cannot induce cough hypersensitivity. It is likely that complex allergic inflammatory reaction in the airway such as interaction between resident and recruited cells, mediators and cytokines is involved in the antigen-induced increase in cough reflex sensitivity as well as LAR. Future studies are required to elucidate the involvement of each possible contributor.

In conclusion, we compared the cough reflex sensitivity to inhaled capsaicin 24 h after an inhaled antigen challenge between actively and passively sensitized guinea pigs. The cough reflex sensitivity and substance P level in BALF were increased in actively sensitized guinea pigs, and further increased 24 h after an antigen challenge. On the other hand, the cough reflex sensitivity or BALF substance P level was not increased after an antigen challenge in passively sensitized animals, while bronchial hyperresponsiveness and airway eosinophilia in BAL were induced by the antigen challenge in both actively and passively sensitized animals. These results suggest that simple IgE- and/or IgG-mediated allergic reaction per se or eosinophilic infiltration in the airway itself may not affect the cough reflex sensitivity or neuropeptide metabolism in guinea pigs, and that cough reflex sensitivity and bronchial responsiveness are modulated by a different mechanism. A complex allergic reaction in the airway may be involved in the development of antigen-induced increase in cough reflex sensitivity.
